# Adaptive Riding: An Intervention to Improve Falls in Individuals with Parkinson’s Disease

**DOI:** 10.1093/geroni/igaf122.4325

**Published:** 2025-12-31

**Authors:** Betsy Kemeny, Whitney Angelini, Chase Decker, Emma Lemire, Alyssa Snyder

**Affiliations:** Slippery Rock University, Slippery Rock, Pennsylvania, United States; Slippery Rock University, Slippery Rock, Pennsylvania, United States; Slippery Rock University, Slippery Rock, Pennsylvania, United States; Slippery Rock University, Slippery Rock, Pennsylvania, United States; Slippery Rock University, Slippery Rock, Pennsylvania, United States

## Abstract

More than 60% of individuals living with Parkinson’s disease fall each year and experience postural instability, gait changes, and fear of falling. Due to both the human-animal bond and activation of the neuromuscular system, equine-assisted services (EAS) are an innovative approach for falls prevention. Both simulated horseback movement and groundwork have shown improved balance in individuals with Parkinson’s disease. No known research focuses on mounted EAS for falls prevention. This pilot waitlist control study measured the effectiveness of six sessions of horseback riding on fall prevention in 12 participants over 60 with Parkinson’s disease using: 1) Tinetti Balance and Gait, 2) Posture Screen App, 3) Adverse Events Questionnaire, 4) Anchored Rating Scale, 5) Activities-Specific Balance Confidence Scale. Findings suggest improvements, trending toward significance, in these specific variables: 1) confidence walking on different surfaces (e.g. ramp, parking lot, mall (p=.06); 2) overall Tinetti score ($\bar{\text{x}}$=22.6, to $\bar{\text{x}}$=24.2), 3) gait [path($\bar{\text{x}}$=1.44, to $\bar{\text{x}}$ =1.78), trunk ($\bar{\text{x}}$=1.11 to $\bar{\text{x}}$=1.44), walking time ($\bar{\text{x}}$=.89 to $\bar{\text{x}}$=1)], 4) balance [chair stand ($\bar{\text{x}}$=1.56,to $\bar{\text{x}}$=1.67), nudged($\bar{\text{x}}$=1.67, to $\bar{\text{x}}$=1.89), step continuity($\bar{\text{x}}$=.67 to $\bar{\text{x}}$=.78), and steadiness($\bar{\text{x}}$=.67 to $\bar{\text{x}}$=1)]. When comparing first to last session, there was significant improvement in maintaining ankle dorsiflexion in saddle (p<.05) and body control (p<.05). Pre to post posture screen comparison suggests improved alignment of the shoulders, hips, and knees. While further research on larger samples with randomized controls are needed, this research demonstrated improvements and feasibility for mounted EAS for older adults living with Parkinson’s disease.

